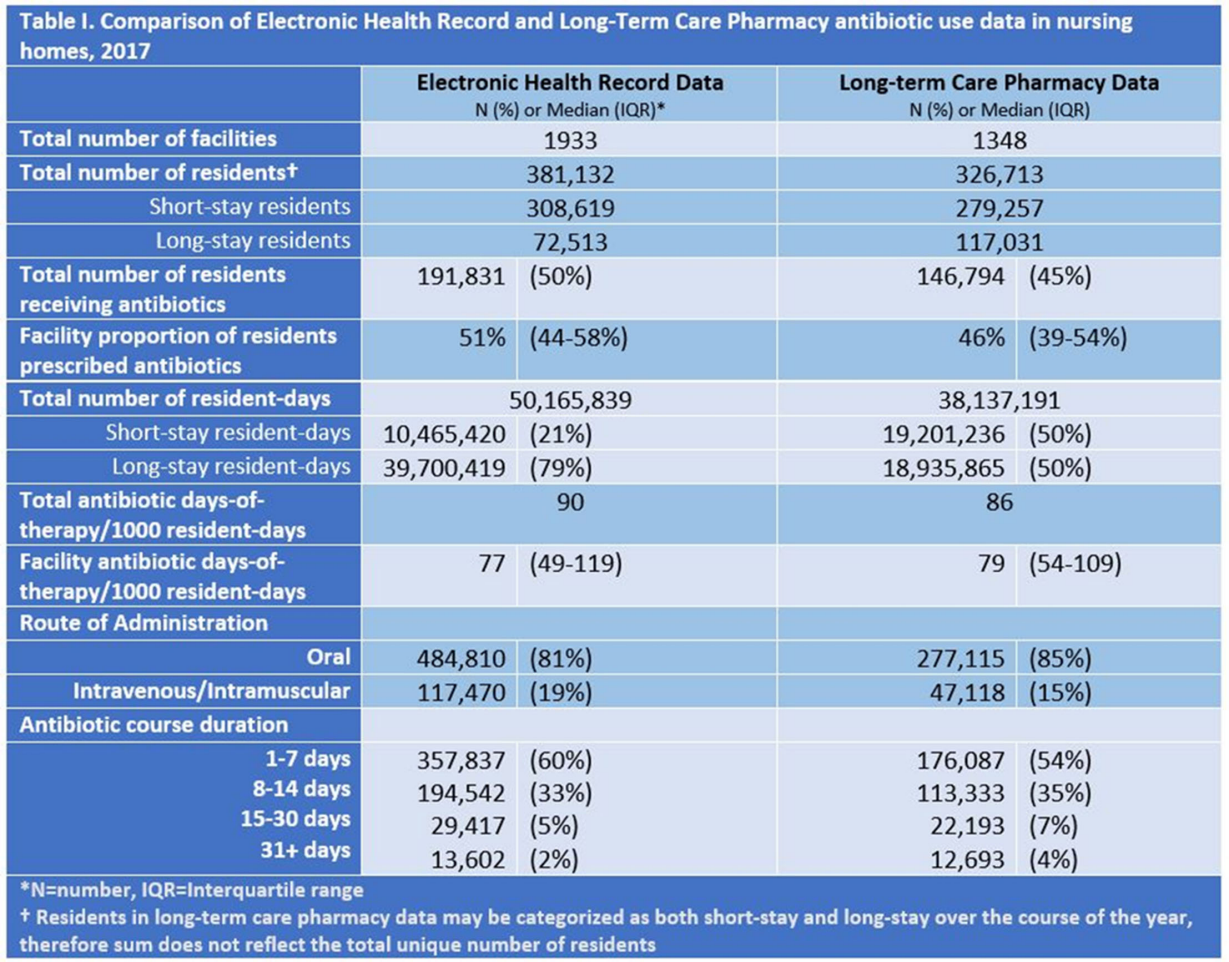# Evaluation of Electronic Health Record and Long-Term Care Pharmacy Data for Tracking and Reporting Antibiotic Use in the United States

**DOI:** 10.1017/ash.2021.24

**Published:** 2021-07-29

**Authors:** Matthew Hudson, Katryna Gouin, Stanley Wang, Manjiri Kulkarni, Mary Beckerson, Laura Ditz, Stephen Creasy, Marti Wdowicki, Nancy Chi, Lauri Hicks, Sarah Kabbani

## Abstract

**Background:** Antibiotics are frequently prescribed in nursing homes, often inappropriately. Data sources are needed to facilitate measurement and reporting of antibiotic use to inform antibiotic stewardship efforts. Previous analyses have shown that the type of nursing-home stay, that is, short stay (<100 days), is a strong predictor of high antibiotic use compared to longer nursing-home stays. The study objective was to compare 2 different data sources, electronic health record (EHR) and long-term care (LTC) pharmacy data, for surveillance of antibiotic use and type of nursing-home stay. **Methods:** EHR and pharmacy data during 2017 were included from 1,933 and 1,348 US-based nursing homes, respectively. We compared data elements available in each data source for antibiotic use reporting. In each data set, we attempted to describe antibiotic use as the proportion of residents on an antibiotic, days-of-therapy (DOT) per 1,000 resident days (RD), and distribution of antibiotic course duration, overall and at the facility level. Facility proportion of short-stay and long-stay (>100 days) nursing-home residents were calculated using admission dates and census data in the EHR data set and a payor variable in the pharmacy data set (Figure 1). The 2 data sources also provided antibiotic characteristics, including antibiotic class, agent, and route of administration. The deidentified nature of facility data prevented direct comparison of antibiotic use measures between facilities. **Results:** The EHR and pharmacy data sets contained 381,382 and 326,713 residents, respectively (Table 1). Within the EHR, 51% of residents were prescribed an antibiotic in 2017, at a median rate of 77 DOT per 1,000 RD. In the LTC pharmacy, 46% of residents were prescribed an antibiotic at a median rate of 79 DOT per 1,000 RD (Table 1). Short-stay residents contributed a smaller proportion of total RDs in the EHR relative to the pharmacy cohort (21% vs 50%, respectively). **Conclusions:** Nursing-home antibiotic use data obtained from EHR and pharmacy vendors can be used for calculating antibiotic use measures, which is important for antibiotic use reporting and facility-level tracking to identify opportunities for improving prescribing practices and provide facility-level benchmarks. Further validation of both data sources in the same facilities is needed to compare antibiotic use rates and to determine the most appropriate proxy for type of nursing-home stay for facility-level risk adjustment of antibiotic use rates.

**Funding:** No

**Disclosures:** None

**Figure 1.**
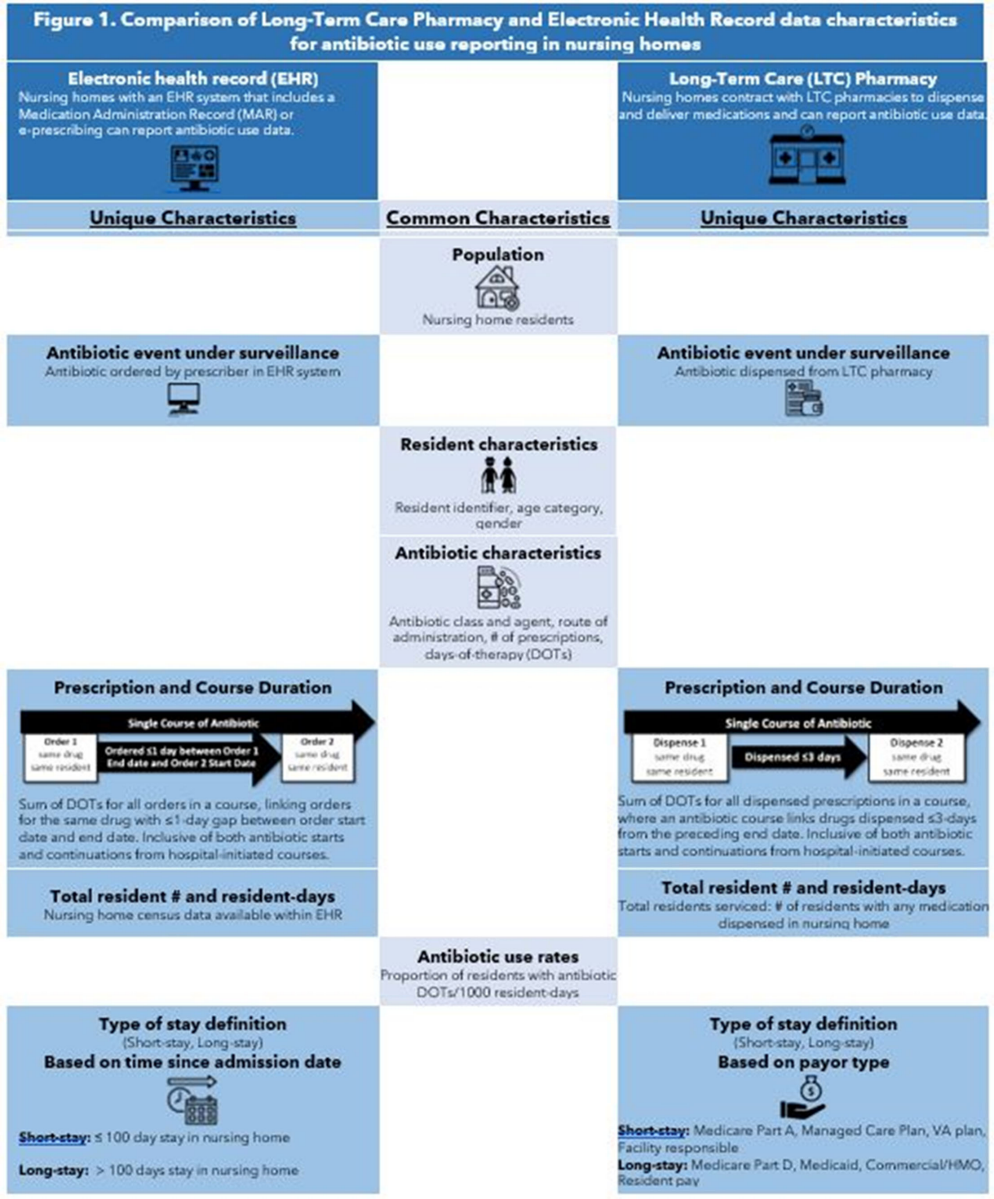

**Table 1.**